# RANBP9 as potential therapeutic target in non-small cell lung cancer

**DOI:** 10.20517/2394-4722.2020.32

**Published:** 2020-06-24

**Authors:** Anna Tessari, Shimaa H. A. Soliman, Arturo Orlacchio, Marina Capece, Joseph M. Amann, Rosa Visone, David P. Carbone, Dario Palmieri, Vincenzo Coppola

**Affiliations:** 1 Department of Cancer Biology and Genetics, College of Medicine, The Ohio State University and Arthur G. James Comprehensive Cancer Center, Columbus, OH 43210, USA.; 2 Department of Medicine, Dentistry and Biotechnology, G. d’Annunzio University of Chieti, Chieti 66100, Italy.; 3 Current address: Department of Biochemistry and Molecular Genetics, Northwestern University Feinberg School of Medicine, Chicago, IL 60611, USA.; 4 Division of Medical Oncology, Department of Internal Medicine, College of Medicine, The Ohio State University and Arthur G. James Comprehensive Cancer Center, Columbus, OH 43210, USA.

**Keywords:** RANBP9, RANBP10, Scorpins, DNA damage, DNA repair, DNA damage response, CTLH complex, cisplatin, non-small cell lung cancer, PARP, BRCAness-like phenotype

## Abstract

Non-small cell lung cancer (NSCLC) remains the leading cause of cancer-related deaths in the Western world. Despite progress made with targeted therapies and immune checkpoint inhibitors, the vast majority of patients have to undergo chemotherapy with platinum-based drugs. To increase efficacy and reduce potential side effects, a more comprehensive understanding of the mechanisms of the DNA damage response (DDR) is required. We have shown that overexpressby live cell imaging (Incuyion of the scaffold protein RAN binding protein 9 (RANBP9) is pervasive in NSCLC. More importantly, patients with higher levels of RANBP9 exhibit a worse outcome from treatment with platinum-based drugs. Mechanistically, RANBP9 exists as a target and an enabler of the ataxia telangiectasia mutated (ATM) kinase signaling. Indeed, the depletion of RANBP9 in NSCLC cells abates ATM activation and its downstream targets such as pby live cell imaging (Incuy53 signaling. RANBP9 knockout cells are more sensitive than controls to the inhibition of the ataxia and telangiectasia-related (ATR) kinase but not to ATM inhibition. The absence of RANBP9 renders cells more sensitive to drugs inhibiting the Poly(ADP-ribose)-Polymerase (PARP) resulting in a “BRCAness-like” phenotype. In summary, as a result of increased sensitivity to DNA damaging drugs conferred by its ablation *in vitro* and *in vivo*, RANBP9 may be considered as a potential target for the treatment of NSCLC. This article aims to report the results from past and ongoing investigations focused on the role of RANBP9 in the response to DNA damage, particularly in the context of NSCLC. This review concludes with future directions and speculative remarks which will need to be addressed in the coming years.

## OUTLINE

As an introduction, we will briefly mention the state-of-the-art strategies in the treatment of NSCLC, as well as the main molecular features of RANBP9. Moving on, we will then succinctly discuss RANBP9 in cancer generally, before focusing on the role of RANBP9 specifically in the cellular response to DNA damage of NCSLC cells.

## INTRODUCTION

### Non-small cell lung cancer

Non-small cell lung cancer (NSCLC) remains the leading cause of cancer-related deaths in the developed world^[1-3]^. The standard of care for NSCLC during the last decades has been the use of combination chemotherapy, including that of platinum-based drugs^[4,5]^. Significant progress has been made in the treatment of this devastating disease through the development of targeted therapies for tumors expressing oncogenic driver alterations (examples include that of *EGFR* mutations and *ALK* rearrangements)^[6-8]^. Nevertheless, the vast majority of NSCLC patients treated with targeted therapy commonly exhibit *de novo* or *acquired* resistance^[7]^.

Treatment with immune checkpoint inhibitors (ICIs) has been transformational in the management of NSLCLC. These drugs have now set new standards to the point that ICIs evolved to become first-line treatment^[9-14]^. However, for unknown reasons, an estimated half of the NSCLC patients are refractory to this new modality and the majority become resistant after an initial response^[15-18]^.

From a clinical and basic science perspective, the relationship between tumor mutational burden and response to ICIs is of great interest^[19]^. It is known that tumors with high mutational burden tend to be immunologically “hot”, displaying favorable responses to treatment^[20]^. Therefore, the specific mechanisms of the DNA damage response (DDR) causing the tumor to be more vulnerable to ICIs represent an active area of research and investigation. Clinical trials testing whether the use of low doses of DNA damaging agents may sensitize advanced NSCLC to targeted or immuno-therapies are ongoing^[21-23]^. Therapeutic regimens include both platinum-based drugs and ICIs for advanced NSCLC, which have shown superior results compared to the use of chemotherapy alone^[21,24]^.Upon first line or after failure of targeted- and/or immune therapies, the vast majority of patients will undergo treatment with platinum-based cytotoxic drugs. Hence, seeking out new modalities of DDR which can be used as biomarkers to better stratify patients, or as new therapeutic targets represents a valuable clinical and experimental goal^[25,26]^.

### Molecular features of RANBP9

**Figure 1.**
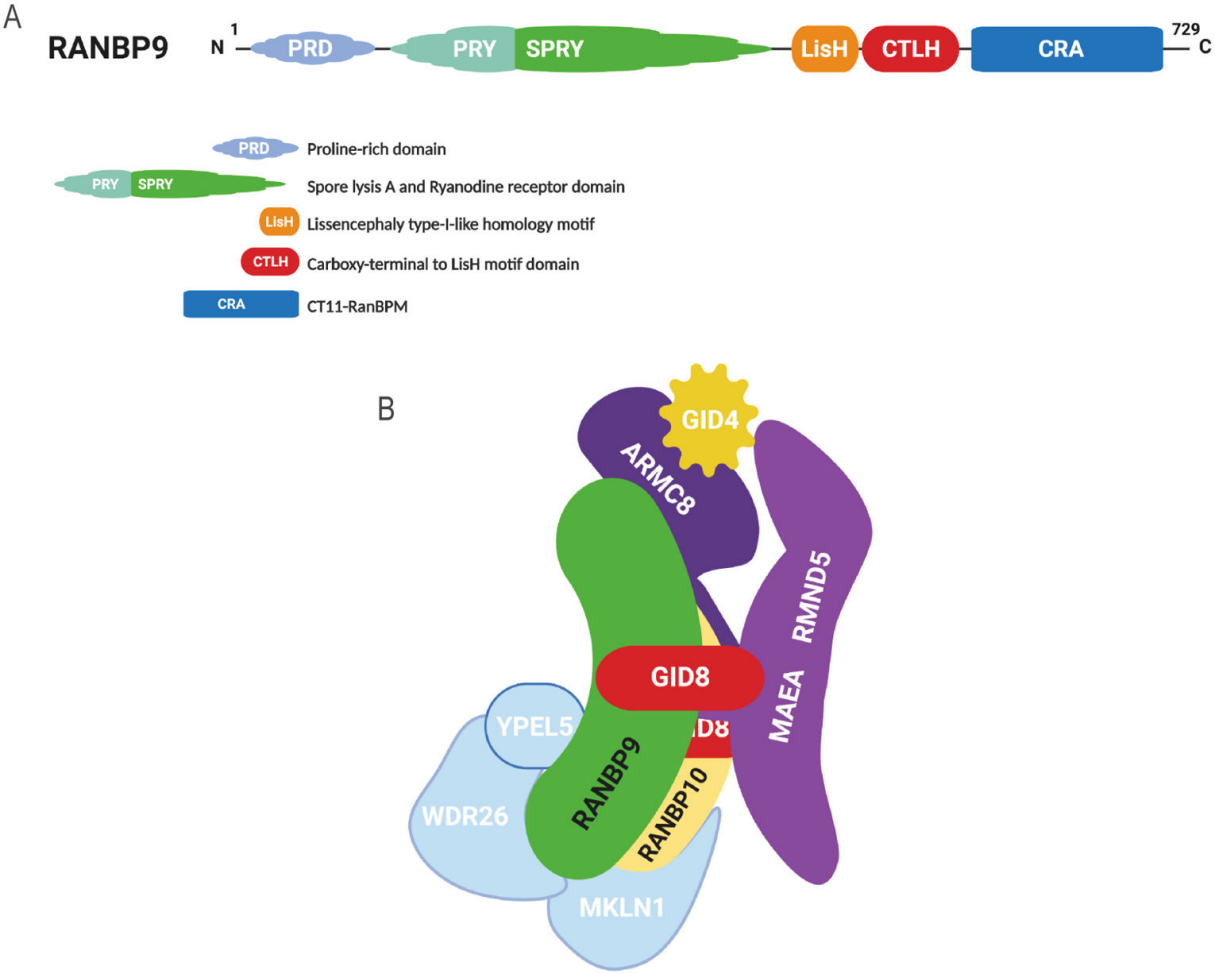
RANBP9 and the CTLH complex. A: RANBP9 is 729 amino acid protein that bears 5 regions/domains that are known to be instrumental for protein-protein interactions; B: the CTLH complex is an evolutionarily conserved E3 ligase multi-subunit structure equivalent of the GID complex in yeast. In its known configuration, the CTLH complex is a heterodecameric structure with a “core” made of a GID8 dimer, RANBP9, and ARMC8 (based on Liu *et al.*^[93]^). Due to the similarities with RANBP9, it is likely that RANBP10 is also a core component. GID4 is a “peripheral” component recognized to act as substrate receptor. Other peripheral CTLH members are MKLN1, WDR26, and YPEL5, whose functions and placement within the structure are not well defined. For more detailed info about the CTLH complex and its members in cancer please refer to Huffman *et al.*^[34]^. RANBP9: RAN binding protein 9; CTLH: C-terminal to LisH domain

The RAN Binding Protein 9 (RANBP9; a.k.a. RANBPM) is a scaffold protein consisting of 5 regions/domains known to mediate protein-protein interactions^[27]^ [Figure 1A]. RANBP9 is highly conserved throughout evolution, suggesting that they maintain critical biological functions^[28,29]^. The perturbation of its expression has shown that RANBP9 modulates the stability, turnover, and consequently signaling, of a number of proteins involved in crucial biological processes/signaling pathways^[30]^. It is thought that RANBP9 exhibits these effects as a component of a ubiquitously expressed multi-subunit structure known as the C-terminal to LisH domain (CTLH) complex, the nomenclature of which is derived from one of the common protein-protein interaction domains shared by most of its members [Figure 1B]^[31-33]^. In its reported configuration, the CTLH complex is heterodecameric, functioning as an unconventional E3 Ligase^[31,32]^. While RANBP9 and GID8 in conjunction make up the scaffold, two other CTLH members known as MAEA and RMND5, provide the enzymatic activity^[31,32]^. Currently, despite limited knowledge the E3 complex as a whole may serve as a key role in cancer biology^[34]^.

### RANBP9 and cancer

With regard to human disease, RANBP9 has been initially studied for its potential involvement in abnormal brain and gonadal development, as well as in Alzheimer’s disease^[35-39]^. Nevertheless, after it has become clear that RANBP9 is linked to critical cancer-causative pathways and to hallmarks of cancer in general, its investigative interest has shifted towards tumor-related paradigms. RANBP9 has been shown to demonstrate tumor-suppressive effects *in vitro*. For example, when acutely over-expressed, RANBP9 is proapoptotic in nature^[40,41]^. Further, it increases stability of other accepted tumor suppressors such as p73 or mammalian Lethal Giant Larvae-1^[42,43]^. Reduced expression of RANBP9 has been associated with distant metastasis and chemo-resistance in gastric cancer^[44]^. The silencing of RANBP9 in colorectal cancer cells also resulted in an increase of cell growth *in vitro* and tumorigenesis *in vivo*^[45]^. However, a straightforward cancer suppressive role is clearly inconsistent with the ability of RANBP9 to enhance Receptor Tyrosine Kinases or MAPK signaling^[46-49]^.

While it remains unclear whether RANBP9’s tumor suppressive effects is observed *in vitro*, most studies agree that RANBP9 is over-expressed in a variety of highly prevalent tumor types including that of NSCLC^[44,45,50-52]^.

### RANBP9 in NSCLC

RANBP9 and the entire CTLH complex are constitutively expressed and respond to different modalities of cellular stress^[31,32,53]^. In order to elucidate the underlying biological mechanisms which could be relevant to the treatment of NSCLC, we focus on studying RANBP9 in the DNA damage response in NSCLC. Our specific interests arose from the observation that γH2AX staining lingered for longer periods of time in RANBP9 knockout (KO) mice testes^[35,54]^. In addition, RANBP9 had been listed as putative target of the ATM kinase in a seminal study by Matsuoka *et al.*^[55]^. In this regard, we previously demonstrated that ATM phosphorylates RANBP9 on at least three Serine residues following DNA damage in NSCLC cells. We also showed that the absence of RANBP9 blunts the effect of ATM signaling^[56]^. This observation prompted us to test the sensitivity of NSCLC cells to DNA damaging agent such as ionizing radiation (IR) and Cisplatin (CDDP), which are frequently used on patients. Our results show that RANBP9 KO NSCLC cells exhibit increased sensitivity to both IR and CDDP^[56]^.

The significance of our findings was highlighted in a retrospective study where we observed that negative correlation existed between the levels of RANBP9 protein expression and response to platinum-based treatment in NSCLC patients^[50]^. As part of the same study, we also determined that the absence of RANBP9 in NSCLC cells caused an increase in the sensitivity to Poly-ADP ribose phosphorylase (PARP)-inhibitors. Recently, our findings were substantiated indirectly by an innovative high throughput CRISPR screening, where the combined ablation of MAEA, UBE2H, and WDR26 (all recognized members of the CTLH complex) was found to be associated with an augmented sensitivity to PARP inhibitors^[57]^. Hence, we speculate that the lack of RANBP9 alone or in combination with other members of the CTLH complex may result in a “BRCAness-like” phenotype in NSCLC^[58]^. This could be clinically important in light of the active pursuit of biomarkers with “BRCAness-like” status in malignancies other than breast and ovarian cancer, including that of NSCLC^[59,60]^.

**Figure 2.**
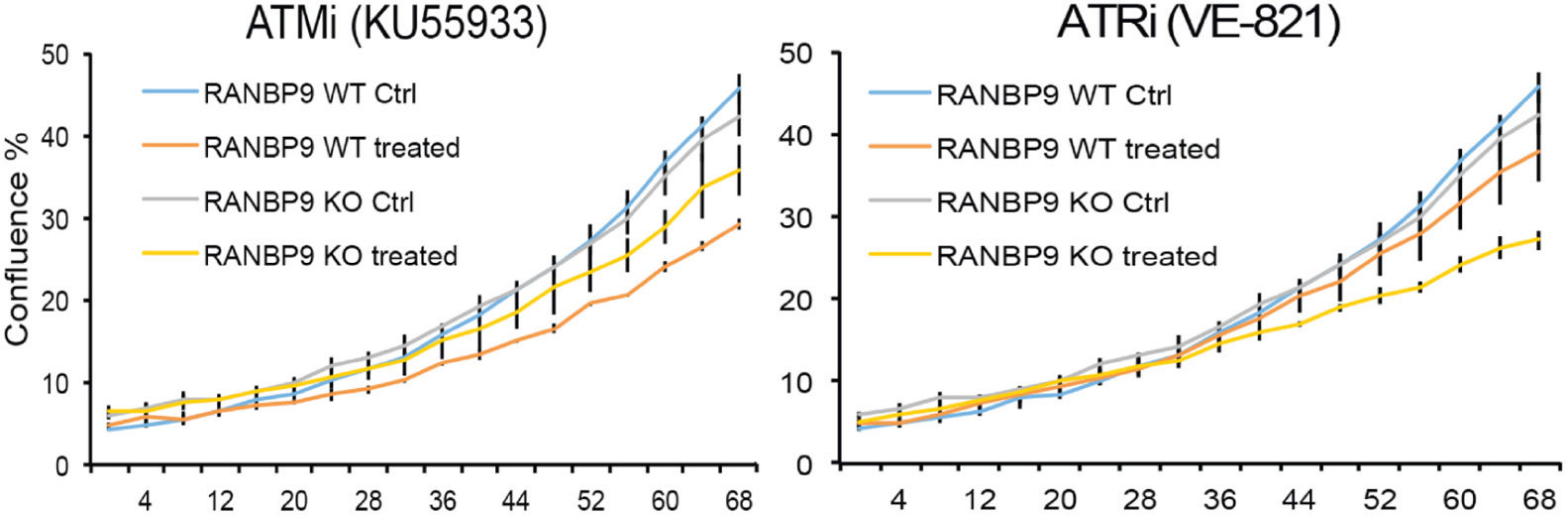
The absence of RANBP9 in NSCLC cells results in increased sensitivity to ATR inhibition. A549 RANBP9 WT controls and A549 RANBP9 KO clones were treated with the ATM inhibitor KU5933 (10 μmol/L; left panel) and the ATR inhibitor VE-821 (10 μmol/L; right panel). Growth was monitored automatically by live cell imaging (Incuyte™) and cell confluence was quantitated in three replica plates. RANBP9: RAN binding protein 9; NSCLC: non-small cell lung cancer; ATR: ataxia and telangiectasia-related; KO: knockout; ATM: ataxia telangiectasia mutated

Herein, we show that RANBP9 KO NSCLC cells have increased sensitivity to ATR inhibitors while not having the same response to ATM inhibitors [Figure 2]. In essence, we hypothesize that RANBP9 levels of expression may be predictive of patient response to specific DNA damaging agents in the clinics. Nevertheless, a prospective study will be necessary to prove this hypothesis^[50]^.

As a whole, RANBP9 is highly expressed in NSCLC compared to normal adjacent tissue^[50]^. However, we have found that the levels of protein expression may not necessarily correlate with the cellular transcription levels. We found this lack of correlation in commonly used NSCLC cell lines as well as in a limited number of freshly extracted NSCLC patient samples^[50]^. This observation needs to be further substantiated and confirmed in other studies. However, it is not unusual for proteins which are part of macromolecular complexes to modulate other proteins’ stability^[61,62]^. In addition, RANBP9 is involved in the response to stress and it is conceivable that protein levels are not always regulated by mRNA expression^[61,62]^. If confirmed, the lack of correlation between RANBP9 protein and mRNA amounts will have profound implications. In fact, it indicates that only the study of protein levels can provide a reliable assessment of the expression of RANBP9.

Taken together, RANBP9 is highly expressed in NSCLC cells as compared to normal lung tissue^[50,51]^. However, this does not preclude the untested possibility that RANBP9 may possess a tumor suppressive function during the initial phases of NSCLC tumorigenesis due to its role in promoting genomic stability. This hypothesis needs to be tested in relevant preclinical models. However, it is conceivable that RANBP9 opposes initiation but may later becomes advantageous for tumor progression, which is similar to TGF-β related signaling pathways^[63,64]^.

## ONGOING INVESTIGATIONS

Studying the role of RANBP9 in the context of cellular response to stress and DNA damage is a major focus of our group. We are currently exploring three specific aspects of RANBP9 biology in response to genotoxic stress. The first is the close association of RANBP9 with the “guardian of the genome” known as p53^[65]^. The second relates to the mechanisms underlying the augmented sensitivity to DNA damaging drugs caused by the lack of RANBP9. Finally, we also consider the potential partial functional redundancy of RANBP10. Due to the presence of high homology, this second Scorpin (Spry-COntaining Ran binding ProteIN) and paralog cannot be ignored, and it is likely a major confounding factor in establishing the importance of RANBP9^[66,67]^.

### RANBP9 and p53 in the DDR

**Figure 3.**
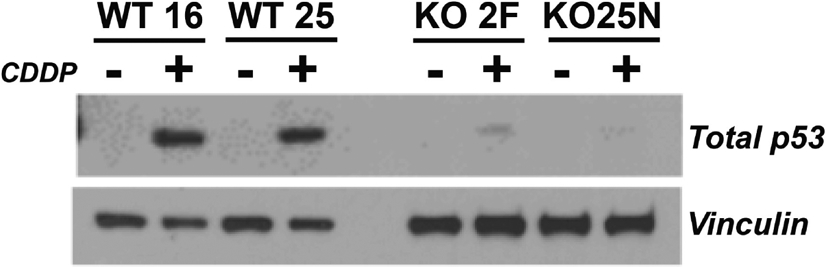
The absence of RANBP9 causes a marked reduction of p53 levels in NSCLC cells subject to genotoxic stress. Two independent A549 RANBP9 WT controls and RANBP9 KO clones were exposed to 10 μmol/L CDDP for 24 h. WB shows that RANBP9 KO cells have a severe reduction of total p53 levels. This is likely due to the blunted p53 phosphorylation of Serine 15, which is a target of the ATM kinase (as shown in Palmieri *et al.*^[56]^, 2016 and Tessari *et al.*^[50]^, 2018). RANBP9: RAN binding protein 9; NSCLC: non-small cell lung cancer; KO: knockout

**Figure 4.**
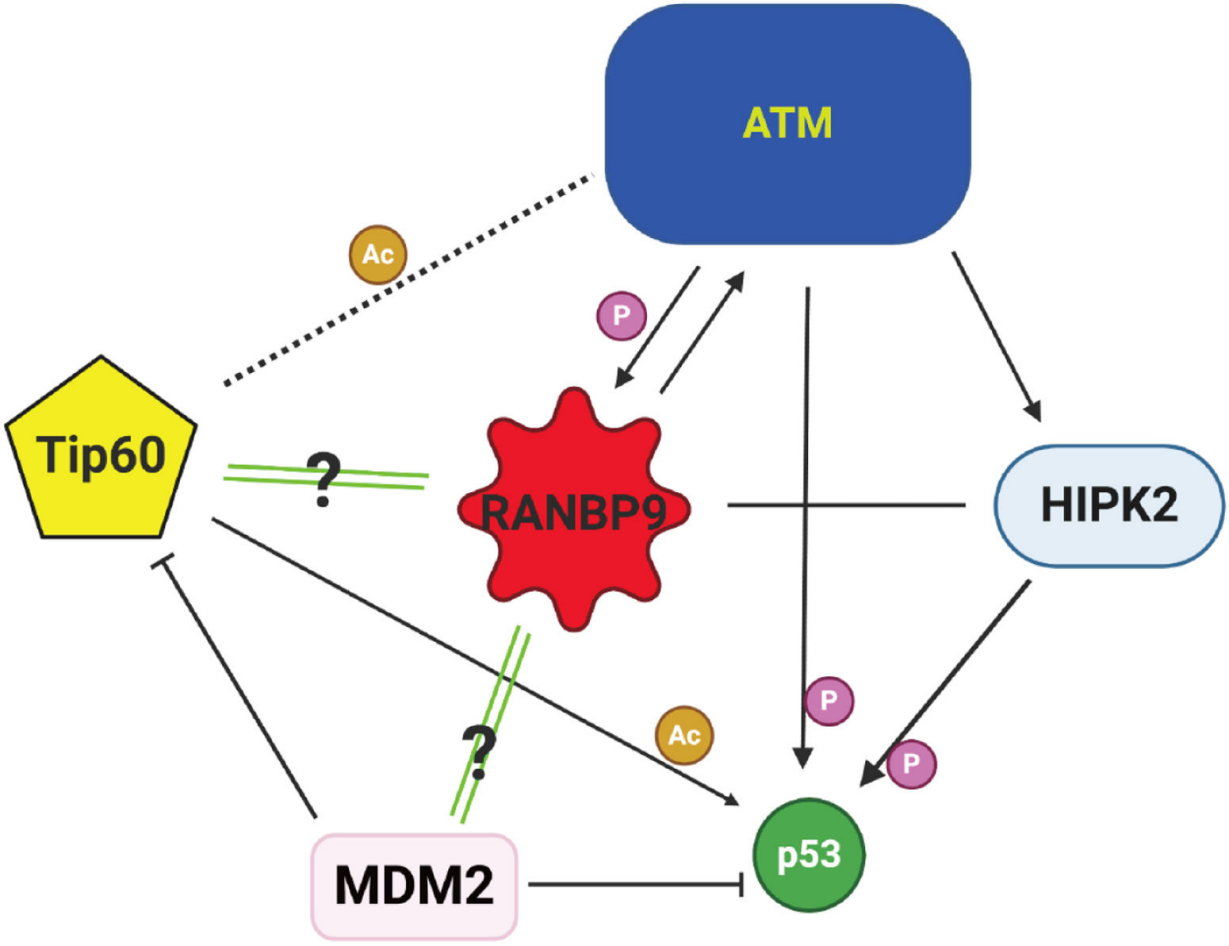
Schematics of the ATM-p53-Tip60-MDM2-HIPK2-RANBP9 potential connection in NSCLC cells subject to genotoxic stress. RANBP9 appears to be at the center of an intricate network determining cell fate during DDR. Green double lines with question marks indicate active areas of investigation. RANBP9: RAN binding protein 9; ATM: ataxia telangiectasia mutated; NSCLC: non-small cell lung cancer; HIPK2: homeodomain-interacting protein kinase 2

As a consequence of impairment of ATM signaling in the absence of RANBP9, we have reported that phosphorylation of p53 on Serine 15 is severely compromised, affecting the total expression of p53 as shown in Figure 3^[50,56]^. The relationship between RANBP9 with p53 is likely more complex than anticipated and worthwhile to be further investigated. Although an interaction has been previously described between RANBP9 and a specific isoform of p73 by co-IP and colocalization, three groups including ourselves have failed to demonstrate a physical interaction between RANBP9 and p53 by co-IP^[42,68]^ (and Coppola, unpublished results). Nevertheless, a high throughput study reported a co-IP between RANBP9 and the p53 R273H mutant that needs to be further validated^[69]^. In summary, it appears that the effects of the absence of RANBP9 on p53 total and phosphorylation levels upon DNA damage are indirect. How the absence of RANBP9 negatively affects ATM-kinase remains to be clarified, but the blunted ATM activity could potentially explain the decrease in p53 levels. On the other hand, an impaired ATM signaling may be only one of the possible mechanisms through which RANBP9 affects p53 abundance and activity. Considering that p53 is degraded by MDM2^[70]^, an unexplored explanation for our findings is that a potential functional link exists between RANBP9 and MDM2. Alternatively, RANBP9 has been reported to co-localize with Tip60 (a.k.a. KAT5)^[71]^. Tip60 mediates histone dynamics in conjunction with PARP1^[72,73]^. Although the acetylation by Tip60 of ATM has recently been put into question^[74]^, it is undisputed that Tip60 acetylates and stabilizes p53^[75]^. It is therefore possible that the absence of RANBP9 further impinges on the stabilization of p53 operated by Tip60 [Figure 4]. Tip60 acetylates p53 on K120, which is crucial for p53-dependent apoptosis^[76]^.

RANBP9 was also reported to have interaction with the homeodomain-interacting protein kinase 2 (HIPK2)^[77]^, causing phosphorylation of p53 on Serine 46 in situations where the DNA damage is beyond repair. Similarly to the K120 acetylation, the phosphorylation of this residue is particularly important in deciding the fate of the cell upon DNA damage and would be in line with the suggested role for RANBP9 in mediating apoptosis^[40,78]^. To complicate the jigsaw regarding the relationship of RANBP9 with ATM, p53, and HIPK2, the latter kinase was degraded via a p53-controlled pathway during recovery from sub-lethal DNA damage^[79]^.

Altogether, RANBP9 appears to be intricately linked to p53 on multiple levels following DNA damage. Therefore, RANBP9 levels play an important role in fine-tuning the activity of the guardian of the genome [Figure 4].

### The absence of RANBP9 confers sensitivity to DNA damage

From a clinical point of view, the absence of RANBP9 renders cells more sensitive to DNA damaging agents such as IR and CDDP, but also to inhibitors of ATR or PARP. This warrants a pre-clinical investigation of RANBP9 as a potential target of therapy which may serve to ameliorate the cancer cell response and resistance to these drugs. However, it is also worthwhile to systematically test RANBP9 KO cells for drug sensitivity and attempt to find additional vulnerabilities caused by the absence of this protein. In fact, RANBP9 is associated with pathways other than the DDR and consequently its absence might result in fatal damage to cancer cells.

### RANBP10 may partially compensate for the absence of RANBP9

To date, the study of RANBP9 has largely ignored the existence of the highly homologous RANBP10, which shares four out of five protein-protein interaction domains^[47]^. The genetic deletion of these two proteins consequently results in two very different phenotypes^[35,36,80]^. However, considering the similarity in protein sequences and genomic organization, they appear to have evolved as duplication of the ancestral yeast Gid1^[28,29]^. We proposed that these proteins may have partially overlapping functions and RANBP10 has been found to be post-translationally modified following DNA damage^[66,81-83]^.

From an experimental perspective, it is conceivable that the similarities between the two Scorpins hinders a clear identification of RANBP9 and nullifies the functional effects of RANBP9 deletion. To test this hypothesis, it will be necessary to perform experiments by deleting both RANBP9 and RANBP10 simultaneously.

Finally, a partial redundancy between the two Scorpins would also explain in part why these genes alone are rarely linked with susceptibility to DNA damaging agents.

Using CRISPR/Cas9, we have recently engineered a novel murine model in which RANBP9 is tagged at the C-terminus with both V5 and HA (RANBP9 with double Tag = RanBP9-TT). This model has been validated through the use of immunohistochemistry and by coimmunoprecipitation, showing that the expression and interactions of the tagged protein faithfully recapitulates those of the wild type RANBP9. This new murine strain will be instrumental in obtaining data *in vivo* about the RANBP9-immunocoprecipated proteome, without the risk of using antibodies that may recognize RANBP10^[84]^.

## CONCLUSIVE REMARKS AND FUTURE PERSPECTIVES

It is clear that treatment of cancer with modalities based on the administration of a single therapeutic agent is rarely successful, whilst combined therapies are more efficacious in the clinical setting. Rationalized drug combinations in anticipation for precision medicine should be developed based on the mechanisms which allow cancer cells to resist and thrive. In particular, RANBP9 has been linked to cell proliferation, cell death, cell adhesion and migration. It has been shown to interact with Receptor Tyrosine Kinases at the membrane, intracellular messengers, and nuclear transcription factors^[30,85]^. In light of these multiple links with critical signaling pathways and critical biological processes, RANBP9 potentially provides a target to block important mechanisms of cell resistance to therapy. Therefore, our group and others have proposed RANBP9 as potential target for cancer therapy^[50,56,85]^. However, this protein is ubiquitously expressed and additional work will be required to take this translational concept to the clinics.

Firstly, we have not fully elucidated the molecular role of RANBP9 is in the DDR. The nuclear accumulation within hours after genotoxic stress suggests the participation of RANBP9 in the resolution of the damage. Apart from being constitutively expressed in the CTLH E3 ligase complex, RANBP9 has also been physically linked to the proteasome^[86]^. Therefore, it is conceivable that it mediates the turnover of proteins that are directly involved in the repair of DNA that need to be disposed of according to the tightly concerted choreography of the DDR^[87,88]^.

With regards to the subnuclear localization of RANBP9 during the DDR, a question which remains is whether RANBP9 is physically present at sites of damage. Novel tools such as the cell and mouse lines with the endogenous RANBP9 tagged may be instrumental in answering this *in-vivo* question.

In addition, Tip60 modulates the acetylation of DNA following cellular damage^[89,90]^. Whether the absence of RANBP9 results in differences in histone acetylation should be a major topic of investigation.

For future clinical management and stratification of patients, a thorough investigation on the outcomes of treatment in the absence of RANBP9 should be conducted using cells containing different mutational status of p53^[91]^. With regard to this, the p53 pathway is the main regulator of cell metabolism^[92-94]^. Recently, RANBP9 has been shown to impinge on crucial metabolic nodes such as AMPK and MTOR signaling including processeses such as autophagy^[53]^. Therefore, the metabolic consequences as a result of the absence of RANBP9 should be investigated to ascertain whether drugs targeting specific metabolic pathways should be used in combination with DNA damaging agents.

In summary, RANBP9 is highly expressed in NSCLC, participating in critical signaling pathways. Therefore, targeting this specific protein may significantly weaken the ability of tumor cells to survive and proliferate when treated with DNA damaging or other types of drugs. Similar consideration may be made with other malignancies in which RANBP9 has been found to be highly expressed.
